# Revisiting Lynam's notion of the "fledgling psychopath": are HIA-CP children truly psychopathic-like?

**DOI:** 10.1186/1753-2000-4-24

**Published:** 2010-09-03

**Authors:** Jared D Michonski, Carla Sharp

**Affiliations:** 1 Department of Psychology, 126 Heyne Building, Houston, Texas, 77204, USA

## Abstract

**Background:**

In his developmental model of emerging psychopathy, Lynam proposed that the "fledgling psychopath" is most likely to be located within a subgroup of children elevated in both hyperactivity/inattention/impulsivity (HIA) and conduct problems (CP). This approach has garnered some empirical support. However, the extent to which Lynam's model captures children who resemble psychopathy with regard to the core affective and interpersonal features remains unclear.

**Methods:**

In the present study, we investigated this issue within a large community sample of youth (*N *= 617). Four groups (non-HIA-CP, HIA-only, CP-only, and HIA-CP), defined on the basis of teacher reports of the Strengths and Difficulties Questionnaire (SDQ), were compared with respect to parent-reported psychopathic-like traits and subjective emotional reactivity in response to unpleasant, emotionally-laden pictures from the International Affective Pictures System (IAPS).

**Results:**

Results did not support Lynam's model. HIA-CP children did not appear most psychopathic-like on dimensions of callous-unemotional and narcissistic personality, nor did they report reduced emotional reactivity to the IAPS relative to the other children. Post-hoc regression analyses revealed a significant moderation such that elevated HIA weakened the association between CP and emotional underarousal.

**Conclusions:**

Implications of these findings with regard to the development of psychopathy are discussed.

## Background

A growing literature has sought to extend the psychopathy construct to youth [1-4]. In one approach to doing so, Lynam [1] proposed locating the future psychopath within the current childhood diagnostic nomenclature. He hypothesized that children high in both hyperactivity, inattention, and impulsivity (HIA), as exemplified in attention deficit/hyperactivity disorder (ADHD), and conduct problems (CP), as exemplified in a diagnosis of oppositional defiant disorder (ODD) or conduct disorder (CD), define a subgroup afflicted with a particularly virulent strain of conduct disorder--what he described as "fledgling psychopathy." In a subsequent test of his model, Lynam [5] found initial support for his predictions. Categorizing a high-risk sample of boys into four groups as a function of their standing on HIA and CP, Lynam found that boys high in both HIA and CP could be reliably distinguished from the other boys (low HIA/low CP, HIA-only, and CP-only) using measures of psychopathic personality, antisocial behavior, and laboratory tasks designed to assess difficulty in delay of gratification and response modulation.

Lynam's [1] model is interesting in that, although he proposes that children elevated in both HIA and CP should most resemble the personality of psychopaths, his model seemingly places little emphasis upon those traits generally regarded as most central to the psychopathy construct [6]. Most definitions of psychopathy draw upon the interpersonal-affective features of psychopathy first described by Cleckley [7] to include characteristics such as superficial charm, egocentricity, dishonesty, shallow affect, and lack of remorse.

Consistent with definitions of psychopathy highlighting diminished affective experience, a number of studies have found reduced emotional reactivity to and processing of negative emotional stimuli in adult psychopaths and in children with psychopathic-like traits. In adults, psychopathy has been associated with reduced autonomic activity to negatively valenced stimuli, as measured via skin conductance (SC) [8] and, perhaps in particular, reduced responsivity to distress cues [9-11]. In child studies, psychopathic-like traits have been associated with reduced SC responses to cues of distress and threat [12], as well as reduced SC to aversive white noise [13]. Furthermore, in adults, psychopathy--specifically the affective and interpersonal features--have been linked to reduced fear response as measured via eye-blink startle reflex, both in criminal [14,15] and community samples [16]. Psychopathic-like traits have also been associated with reduced cognitive-affective processing of negative emotional stimuli, as evidence by reduced attentional orienting to negative emotional words [17,18] and distressing pictures [19], as well as reduced recognition of facial displays of distress [20,21], sad vocal tones [21], and self-report of reduced emotional reactivity in response to emotionally intense and unpleasant pictures [22].

In contrast, Lynam's method for locating the fledgling psychopath (HIA-CP) appears to place primary emphasis upon the impulsive, irresponsible, stimulation-seeking (behavioral) dimension of psychopathy. An important question, however, is whether children high in HIA and CP exhibit the characteristic affective and interpersonal features of psychopathy. Research has well established that the combination of HIA and CP constitutes a particularly at-risk subgroup of aggressive youth, more so than HIA-only or CP-only children [23,24]. However, few studies have explicitly tested Lynam's [1] prediction that HIA-CP children look the most psychopathic in terms of their personality. In his empirical investigation of his model, Lynam [5] found that the HIA-CP group was higher than the other groups in psychopathic-like traits, as measured by mother reports on the Childhood Psychopathy Scale (CPS), but the difference relative to the CP-only group was nonsignificant. Barry et al. [25] employed Lynam's groupings, designating a group of children who met criteria for ADHD and ODD/CD, another who met criteria for ADHD but not for ODD/CD, and a third group consisting of clinic-referred controls who did not meet criteria for ADHD or ODD/CD (an ODD/CD only group was not included due to insufficient sample size). Of note, Barry et al. [25] compared the groups of children on teacher reports of the core affective/interpersonal features of psychopathy and found that the ADHD-ODD/CD group had a significantly higher proportion of children elevated on callous-unemotional (CU) traits than both the ADHD only and clinical control group. Retrospective reports of adult psychopaths also provide support for Lynam. Johansson, Kerr, and Andershed [26] found that psychopathic criminals were considerably more likely than non-psychopathic criminals to report childhood histories of CP and HIA.

Further support for Lynam's approach comes from studies considering the specific role of ADHD in promoting psychopathic traits. Fowler et al. [27] found that their sample of ADHD children exhibited higher total psychopathic traits, and affective traits (as measured by the PCL-R [28]) in particular, compared to a community sample of adolescents. Piatigorsky & Hinshaw [29] reported similar findings: ADHD boys were significantly more psychopathic-like than control boys. This difference remained even after controlling for ODD status, indicating that ADHD exerted a unique effect upon psychopathy ratings.

These studies provide some initial support for Lynam's [1] proposal. However, the number of attempts to test Lynam's model is still relatively few. In the present study, we seek to revisit Lynam's model for locating the fledgling psychopath in the current childhood diagnostic nomenclature. In particular, we are interested in how Lynam's group designations compare with respect to the affective/interpersonal features of the construct. If Lynam's model is sufficient to identify children most resembling adult psychopaths, then the following hypotheses should be supported: First, children elevated in teacher-reported HIA and CP should be rated by parents as more psychopathic-like on the Antisocial Process Screening Devise (APSD) [30] than comparison groups (no problems, HIA-only, and CP-only). More specifically, the HIA-CP group should exhibit greater affective/interpersonal (callous-unemotional) traits, not just elevated scores on the total or impulsivity dimension. Secondly, HIA-CP children should report reduced emotional reactivity in response to unpleasant emotionally-laden pictures (International Affective Picture System) [31] than the comparison groups. Given that this study employed multiple sources of report (parent report of psychopathic traits, teacher report of conduct and hyperactivity problems, and self-report of emotional reactivity) support for the above hypotheses cannot be attributed to shared method variance and is therefore a strength of the current study.

## Methods

### Participants

The present study was a part of a larger investigation of social-cognitive and emotional correlates of antisocial behavior in a large community sample of children (the Child Behavior Study). Participants consisted of 2,950 7 to 11 year-old children recruited from 16 primary schools in Cambridgeshire, England. Of the parents contacted, an average of 22% granted consent for their child's participation. The response rates for individual schools ranged from 14 to 40%. A total of 659 children (319 boys and 340 girls) were initially enrolled. After removal of children with incomplete data, this number was reduced to 617 in the current analysis.

In order to examine the generalizability of findings, the school board's permission was asked so that teachers from one of the schools anonymously completed a population-based screen of psychiatric problems (the Strengths and Difficulties Questionnaire (SDQ) [32]) on all children, allowing for comparison of children whose families did not consent to participate with those who did. Of note, no significant differences were present across all five subscales of the SDQ. Secondly, comparison of sociodemographic characteristics of the sample of participants to regional statistics revealed no evidence of selection bias. The ethnic distribution in the sample was in line with regional statistics for Eastern England (97% White, 2% Asian, 0.5% Black and 0.5% Oriental) and comprised of 40% wealthy achievers, 9% urban prosperity, 28% comfortably well-off, 9% moderate means, and 14% hard pressed. The mean age and IQ for children participating in the present study was 9.6 (*SD *= 1.22) and 104 (*SD *= 17), respectively.

### Measures

#### Teacher-reported hyperactivity and conduct problems

Parents and teachers completed the Strengths and Difficulties Questionnaire (SDQ) [32]. The SDQ is a 25-item behavioral screening measure that was designed to provide a brief assessment of child psychiatric disorders in children ages 3 to 16. Despite its brevity, the SDQ has been shown comparable to the much longer Child Behavior Checklist (CBCL) [33] in assessing internalizing and externalizing problems and may be better than the CBCL in detecting inattention and hyperactivity [34]. The SDQ produces five subscales, four of which measure psychopathology: emotionality, conduct problems, hyperactivity/inattention, and peer problems. The remaining subscale measures prosocial behavior. The five subscales demonstrate adequate internal reliability, particularly teacher report (used in the current study). Cronbach's α has been found to range from .70 (peer problems) to .88 (hyperactivity/inattention) for teacher report [35]. In the current study, Cronbach's α was .89 (hyperactivity/inattention) and .73 (conduct problems) for the two teacher-reported subscales used. The SDQ has been shown to accurately detect psychiatric diagnoses in community [36,37] and clinical samples [38]. It has demonstrated a specificity of 94.6% (95% CI 94.1-95.1%) and a sensitivity of 63.3% (59.7-66.9%) in identifying psychiatric diagnosis, and performed particularly well with regard to conduct-oppositional disorders and hyperactivity disorders (sensitivity ranging from 70-90%) [36]. Due to its success, it has now been translated into over 60 languages and it is being used all over the world.

#### Parent-reported psychopathic personality traits

The Antisocial Process Screening Device [30] is a 20-item behavioral rating scale used to assess psychopathic-like traits in youth. Factor analytic studies have generally revealed three dimensions: a 7-item narcissism factor, a 5-item impulsivity factor, and a 6-item callous-unemotional (CU) factor, with moderate correlations among the factors [39-41]. Frick et al. [40] reported internal consistencies ranging from .74 (impulsivity) to .83 (narcissism); however, subsequent studies have typically found lower internal reliabilities, especially for the CU subscale [39,42]. Cronbach's α for parent reports in the present study was .81 for the total APSD, with values of .52 (CU), .64 (impulsivity), and .67 (narcissism) for the subscales. One promising approach to improve the psychometric properties of the CU scale that has been used in a number of studies [e.g., [39,43,44]] combines items from the prosocial behavior scale of the SDQ with the best-performing items of the APSD CU scale [see [39]]. We adopted this approach to measure CU traits in the present study. Specifically, CU traits were computed as the sum of three APSD CU items (unconcerned about others' feelings; lack of guilt; breaks promises) and five SDQ Prosocial items, reverse coded (considerate of other's feelings; shares with other children; helpful if someone is hurt, upset, or ill; kind to younger children; volunteers to help others). This composite CU scale improved the internal reliability to .78 in the present sample.

#### Emotional reactivity

To measure subjective emotional reactivity, the International Affective Pictures System (IAPS) [31] was used. The IAPS has a long tradition in the adult literature and has recently been applied to samples of young children [e.g. [22,45,46]] based on norms proved by Lang and colleagues for the 7-11 year age range [31]. These studies have demonstrated validity for this measure in young samples for use in community studies [45] and in relation to psychopathic-like traits and conduct problems [22]. For instance, in the original validation study of the IAPS as used in the current study, Sharp, Van Goozen and Goodyer [22] showed that the IAPS could elicit similar responses across a wide range of affective content and with similar gender differences as previously found in adults.

The same 27 pictures used in the Sharp, Petersen and Goodyer [46] study were used here. Pictures varied with respect to valence and arousal. All pictures were mounted as A4 photographs in color, with high figure/ground contrast in order to ease discrimination of relevant features. Pictures were selected for age-appropriateness. In keeping with research that investigates reduced emotional reactivity associated with externalizing disorders [22], only high arousal/negatively valenced pictures were considered in the present study. Although valence and arousal were both rated for these pictures, only arousal ratings were used for analyses, as valence ratings showed no relation to psychopathic-like traits. To subjectively report their emotional reactions, children used a paper-and-pencil version of the Self-Assessment Manikin (SAM) [47]. This is a child-friendly approach that enables children to make dimensional ratings of arousal on a 9-point scale with 1 indicating low arousal and 9 indicating high arousal. This approach has been shown to yield valid responses in children [22,45]. For determining indices of arousal, we followed standard convention in using IAPS subjective ratings [22,45] and calculated the mean of arousal ratings to unpleasant, pleasant and neutral pictures respectively.

#### IQ

A shortened version (Vocabulary and Block Design) of the Wechsler Intelligence Scale for Children [48] was individually administered to children. This shortened method has been validated to be an adequate measure of IQ [49]. Sattler's [49] guidelines were used to score the measure.

#### Socio-economic status

To determine socio-economic status, we used a geodemographic tool called ACORN which is freely available on the internet. ACORN categorizes all 1.9 million UK postcodes, which have been described using over 125 demographic statistics within England, Scotland, Wales and Northern Ireland, and 287 lifestyle variables, making it a powerful discriminator for social class. For our purposes we used ACORN's 5-class system to determine membership to one of the following: 0 for Wealthy Achievers, 1 for Urban Prosperity, 2 for Comfortably Well Off, 3 for Moderate Means and 4 for Hard Pressed.

### Procedures

The first step in recruitment and consent procedures involved contacting head teachers in the Cambridge area. For those head teachers who consented, information packets and consent forms were delivered to be passed on to children and parents. Our research team did not have access to names and contact details of parents or children prior to consent. Postal informed consent was obtained from all parents and child assent was obtained in person prior to data collection. Children and parents were told that the study was about understanding behavior problems in children, and the factors that may influence behavior problems in children. Since the Child Behavior Study focuses mostly on social cognitive and affective processing correlates of antisocial behavior, children were told that the study was about understanding behavior problems and how thinking and feeling affected behavior. Approval was also sought and obtained from the local ethics committee prior to data collection.

The administration of the IAPS and IQ testing took place individually in a quiet room at school with adequate lighting. The IAPS photographs were mounted on a stand and shown for 10 seconds with 10-second intervals between photographs. As suggested by the manual, children were trained to use the SAM on a practice trial. Following McManis et al.'s [45] work with pre-adolescent children, words like happy, pleased, or good, and unhappy, scared, angry, bad or sad were used in the instructions to describe the endpoints of the pleasure (valence) scales. Words like calm, relaxed, bored, or sleepy and excited, nervous or wide-awake described the endpoints for the arousal scale.

Teachers were consulted as to the level of understanding for the 7-year-olds (youngest cohort) of all questionnaire measures, and it was decided that questions would be read aloud to this group for the self-report measures. Care was taken not to influence children's answers in any way. Children in higher grades were invited to ask for help if needed. However, none of the children in the high grades did so. Questionnaires were administered individually and in private with children in an empty classroom. Parent reports were obtained through mail. Teacher report was obtained during the period of assessment in a particular school.

### Group Designation and Data Analytic Strategy

Groups were formed on the basis of teacher reports on the hyperactivity (HIA) and conduct problems (CP) scales from the SDQ. The clinical cutoffs developed and normed by the developers of the SDQ http://www.sdqinfo.com were used to identify children high in HIA and/or CP. Means and standard deviations for teacher reports of hyperactivity and conduct problems for each of the four groups appear in Table 1. Each group differed significantly from one another in hyperactivity and conduct problems. As to be expected, both HIA groups were rated as more hyperactive than the CP-only and non-HIA-CP groups. Similarly, both CP groups were rated as exhibiting more conduct problems than the HIA-only and non-HIA-CP groups. Groups were also compared on variables that have been shown to correlate with externalizing problems (age, gender, and IQ). Because groups differed with respect to gender composition and IQ, these variables were considered as covariates.

**Table 1:** Mean Scores of Four Comparison Groups on Demographic and HIA-CP Variables

	*Non-HIA-CP* (*n *= 517)		*HIA-only* (*n *= 48)		*CP-only* (*n *= 23)		*HIA-CP* (*n *= 29)	
Variable	*M*	*(SD)*	*M*	*(SD)*	*M*	*(SD)*	*M*	*(SD)*
Demographics								
Age	9.60	(1.20)	9.37	(1.16)	9.73	(1.29)	9.36	(1.27)
Gender (% male)	45.26^a^		62.50^b^		52.17^a, b^		65.52^b^	
IQ	106.30^a^	(16.13)	97.90^b^	(19.58)	101.35^a, b^	(20.24)	92.81^b^	(14.22)
SDQ (Teacher)								
Hyperactivity	1.66 ^a^	(1.91)	8.10^b^	(0.99)	3.52^c^	(2.08)	8.97^d^	(1.12)
Conduct Problems	0.42^a^	(0.80)	1.71^b^	(0.99)	4.39^c^	(0.58)	5.52^d^	(1.62)

*Note*. Means with different subscripts differ significantly at p < .05. SDQ = Strength and Difficulties Questionnaire.

To test each hypothesis, we conducted a set of three planned, pairwise comparisons, whereby each group was compared to the HIA-CP group. The first contrast (*Cont. 1*) tested the non-HIA-CP group against the HIA-CP group; the second contrast (*Cont*. 2) tested the HIA-only group against the HIA-CP group; and the third contrast (*Cont. 3*) compared the CP-only group against the HIA-CP group. Type I error rate was maintained at α = .05 for testing each dependent variable using Dunnett's procedure.

## Results

### Psychopathic Traits

To test our first aim of whether the subgroup of conduct problem children identified by Lynam constitutes the "fledgling psychopath," we compared the four groups on parental ratings of child psychopathic personality traits (APSD). As Table 2 displays, planned contrasts revealed that the HIA-CP group appeared the most psychopathic-like with respect to parental report of APSD total score. The HIA-CP group was rated as significantly higher than all other groups, albeit the difference between the CP-only and HIA-CP groups reached significance only for a 1-tailed test^1^.

**Table 2:** Mean Scores on Measures of Psychopathic Traits and Emotional Reactivity to Unpleasant Pictures by HIA-CP Group Designation

	*non-HIA-CP*		*HIA-only*		*CP-only*		*HIA-CP*		*t-values*		
Variable	*M*	*(SD)*	*M*	*(SD)*	*M*	*(SD)*	*M*	*(SD)*	*Cont. 1*	*Cont. 2*	*Cont. 3*
Psychopathy (Total)	8.40	(4.49)	10.42	(4.13)	12.12	(5.30)	15.50	(6.95)	6.72***	3.88***	2.22^†^
Narcissism	2.24	(1.97)	2.30	(1.65)	3.18	(1.59)	4.20	(2.91)	4.32***	3.37**	1.56
Impulsivity	3.64	(1.82)	4.85	(1.68)	4.76	(1.75)	6.20	(2.19)	6.15***	2.62*	2.39*
Callous-Unemotional	3.00	(2.56)	3.87	(2.64)	4.27	(3.03)	5.53	(3.79)	3.88***	2..08^†^	1.35
Arousal to Unpleasant Pictures (IAPS)	5.66	(2.28)	4.87	(2.56)	3.88	(2.63)	4.74	(2.78)	1.95	< 1	1.28

*Note*. Total, Narcissism, and Impulsivity score come from parental report of the Antisocial Process Screening Device (APSD). Callous-Unemotional is a composite of items from parent reports of the APSD callous-unemotional traits scale and the Strength and Difficulties Questionnaire (SDQ) prosocial behavior scale. HIA (Hyperactivity) and CP (Conduct Problems) come from teacher report on the SDQ. IAPS = International Affective Pictures System. Cont. 1 = contrast comparing mean *non-HIA-CP *to *HIA-CP*; Cont. 2 = contrast comparing mean *HIA-only *to *HIA-CP*; Cont. 3 = contrast comparing mean *CP-only *to *HIA-CP*. Type I error was maintained at α = .05 for all pairwise comparisons using Dunnett's procedure. *t-*values are based on 486 (APSD), 456 (Callous-Unemotional), and 599 (IAPS) degrees of freedom.

* p < .05; ** p < .01; *** p < .001; ^† ^1-tailed test, p < .05.

Specific hypotheses were also tested with regard to the narcissism, impulsivity, and CU subscales. We were particularly interested in testing whether Lynam's fledgling psychopathy group would be rated highest on the core affective/interpersonal traits of psychopathy (CU subscale). As shown in Table 2, the HIA-CP was consistently higher than the other groups across each subscale, but not all of these differences reached significance. The HIA-CP group was significantly higher than the non-HIA-CP and HIA-only groups for narcissism, impulsivity, and CU traits. However, the contrasts between the HIA-CP and CP-only groups revealed that the higher scores for the HIA-CP group were significant only for the impulsivity subscale^2^.

### Emotional Reactivity to Unpleasant Pictures

Conceptually, both CU traits and reduced reactivity to negative emotional stimuli are important indicators of psychopathy. Thus, one would expect the two criteria to be significantly associated--specifically, to be negatively correlated--such that higher CU traits correspond to decreased arousal ratings. As expected, parent-report of CU traits and self-reported arousal ratings to the negative emotional pictures were significantly negatively correlated, albeit the effect was small (*r *= -.10, *p *< .05).

To test our second aim, we compared the four groups on subjective emotional reactivity to unpleasant pictures. As shown in Table 2, and contrary to expectations, none of the planned contrasts examining group differences in relation to the HIA-CP group were significant. HIA-CP children were not found to report experiencing the lowest degree of arousal in response to the pictures. Rather, the CP-only group reported less arousal than did the HIA-CP group. When considering the effect of CP regardless of standing on HIA, children elevated in CP traits reported significantly lower arousal to unpleasant pictures than did children low in CP: *F*(1, 206) = 12.51, *p *< .001.

**Figure 1:**
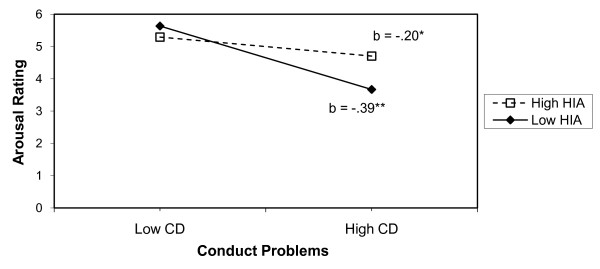
**Interaction of Conduct Problems and Hyperactivity on Arousal Ratings**. *Note*. HIA (Hyperactivity) and CP (Conduct Problems) come from teacher report on the Strengths and Difficulties Questionnaire. High and low conditional values for the predictors represent the 90th and 50th percentiles, respectively. * p < .05; ** p < .01.

These findings raised the possibility that HIA was actually functioning to protect children high in CP against reduced emotional reactivity. In order to further explore this possibility, we conducted an exploratory hierarchical regression analysis to consider whether HIA may moderate the relation between CP and arousal rating. In step 1, HIA and CP were entered into the model. In step 2, the interaction term (HIA×CP) was entered. Both predictor variables (HIA and CP) were centered in order to reduce nonessential multicolinearity [50]. As shown in Table 3, results revealed that CP emerged as a significant predictor of arousal (*B *= -.25, *p *< .05), while HIA did not (*B *= -.05, n.s.). Of greater interest, the interaction term (Step 2) was also significant (*B *= .04, *p *< .05), albeit the effect size for the model was small (*R^2 ^*= .05). Even so, the direction of the moderation effect is interesting, as revealed by the plotting of the interaction (Figure 1). Probing and plotting of the interaction followed the conventions recommended by Aiken and West [50]. For testing of simple slopes, high and low conditional values of the predictors were chosen as the 90^th ^and 50^th ^percentile, respectively. The simple slope of arousal regressed on CP was significant at both levels of HIA. As depicted in Figure 1, the magnitude of the negative association between CP and arousal ratings was weakened at higher levels of HIA^3^.

**Table 3:** Hierarchical Regression of Arousal to Unpleasant Pictures on HIA and CP

	*B*	***sr***^**2**^	***R***^**2**^
Step 1			.04
HIA	-.05	.00	
CP	-.25*	.02	
Step 2			.05
CP×HIA	.04*	.01	

*Note*. HIA (Hyperactivity) and CP (Conduct Problems) come from teacher report on the Strength and Difficulties Questionnaire (SDQ).

* p < .05.

## Discussion

The objective of the current report was to revisit Lynam's [1,5] operationalization for capturing the fledgling psychopath. This model contends that the future psychopath is most likely to emerge from within a subgroup of children elevated in both HIA and CP. In keeping with Lynam [5], we tested this model by comparing children designated into four groups based on their status with respect to HIA and CP. Specifically, we were interested in whether high HIA-CP children would resemble the adult psychopath in terms of the core affective and interpersonal personality features associated with psychopathy. These features were assessed using parental reports of psychopathic-like traits and using subjective emotional reactivity in response to unpleasant, emotionally-laden pictures. As such, the results of our study cannot be attributed to shared method variance.

Overall, we did not find support for Lynam's model. The primary dimension of psychopathic-like traits by which the HIA-CP children were distinguished from the other groups was impulsivity. However, with regard to callous-unemotional and narcissistic traits, the HIA-CP was indistinguishable from the CP-only group. Thus, HIA-CP children did not appear the most psychopathic-like in terms of the core affective and interpersonal traits. The link with impulsivity is consistent with Lynam's [5] findings on laboratory tasks. Lynam found significant differences between the HIA-CP and CP-only groups on tasks involving delay of gratification and a neuropsychological task requiring attention and concentration to perform sequences of complex behaviors. Impulsivity may be *an *important dimension of psychopathy [51,52]. However, it is doubtful that anyone would argue for the sufficiency of impulsivity in distinguishing youth at risk for psychopathy.

Further difficulty for Lynam's model emerged with respect to emotional reactivity to unpleasant pictures. Namely, the HIA-CP children did not self-report experiencing the lowest level of emotional reactivity, as would be anticipated if they were truly the most psychopathic-like. In fact, the HIA-CP group did not differ significantly from any of the other groups. When the association among HIA, CP, and emotional reactivity was explored using a regression approach, HIA actually showed a softening effect upon the relation between emotional arousal/reactivity and CP, as evidenced by a significant HIA by CP interaction. High levels of HIA appeared to protect high CP children from exhibiting affective underarousal.

Other studies have reported a similar effect for HIA. In a study of incarcerated adolescents, Sevecke, Kosson, and Krischer [53] examined the effect of ADHD and CD symptoms upon psychopathic traits, assessed using the Psychopathy Checklist-Youth Version (PCL-YV). Interestingly, for boys, although ADHD did exhibit significant bivariate relations with the PCL-YV total and four dimensional scores (interpersonal, affective, lifestyle, and antisocial), these effects remained significant only for the antisocial dimension when CD was added to the regression model. Additionally, with the exception of antisocial traits, no synergistic effects were observed for ADHD and CD in predicting psychopathic traits^4^. In another study, Loney et al. [17] examined the impact of both CU traits and impulsivity upon the processing of emotionally-laden words in a sample of low to moderate at-risk youth. They found that, while CU traits were associated with slower reaction times for recognition of negatively valenced words, HIA was associated with faster recognition of negatively valenced words. These finding do not suggest a prominent role for HIA symptoms in contributing to what many regard as a crucial component of psychopathy--i.e., deficient affective experience [6,7,54]. In Loney et al. [17], results suggested that HIA is actually associated with greater reactivity to negative emotional stimuli--a finding which would appear to run counter to reports in which psychopathy has been associated with decreased responsiveness to negatively valenced emotional stimuli [12,14].

The present study, along with these prior findings [17,53], calls into question the utility of prioritizing the combination of HIA and CP for delineating a subgroup most at risk for emerging psychopathic personality. The fact that some studies have demonstrated a more persistent pattern of antisocial behavior in youth with comorbid ADHD and CD appears therefore to be primarily due to the increased levels of conduct problems in this comorbid group, rather than the influence of ADHD symptoms per se [6]. An alternative and more direct approach to capturing a childhood analogue of adult psychopathy makes the affective and interpersonal features more central (for reviews see [6,52,55,56]). Barry et al. [25], for instance, found that, in addition to HIA and CP, CU traits were necessary to distinguish a subgroup of psychopathic-like children who showed a preference for thrill and adventure-seeking activities and exhibited a reward-dominant response style on laboratory tasks. Their findings suggested that Lynam's subtyping approach may designate an overly broad, perhaps heterogeneous subgroup of children [57], especially against the background of studies showing that a substantial number of children with childhood-onset conduct problems also exhibit co-occurring ADHD [58]. Therefore, this subtyping may not designate a group that is very distinct from the broader group defined by an early age of onset [59]. In contrast, there is now an impressive body of evidence to suggest that the interpersonal and affective features originally described by Cleckley [7] as the hallmark of the psychopathic personality may better delineate a subgroup of antisocial youth resembling the "fledgling psychopath."

Another possibility is that the model advanced by Lynam [1] is more inclined to identify children at risk for developing a different form of antisocial/psychopathic personality from the traditional conceptualization [7]. Psychopathy has a long history of being viewed as consisting of various forms and subtypes. Karpman [60] was first to distinguish between primary and secondary psychopathy. Primary psychopaths are more in keeping with the Cleckleyian view of psychopaths as cold, callous, manipulative, and egocentric, whereas secondary psychopaths are viewed as neurotic and impulsive, their antisocial behavior stemming from emotional conflict. Perhaps Lynam's HIA-CP children are at greater risk for this latter type.

### Strengths and Limitations

Our study presents several limitations that should be noted. For one, the controversial practice of applying the label of "psychopathy" to children [61,62], especially a community sample of children, deserves comment. In line with other studies of psychopathy in community samples [39,63-65] we operationalize psychopathy as traits that lie on a continuous dimension, as opposed to a categorical diagnosis. Research in community samples is important because it provides the opportunity to identify developmental pathways by which psychopathy may develop in children and adolescents. Indeed, several recent reviews of the psychopathy literature [57] have called for more research in community samples. Notwithstanding the advantages of using community studies in this line of research, the number of children above cut-off for conduct problems, hyperactivity, and both combined was relatively small. For instance, less than 5% of participants fell into the HIA-CP category. It is therefore important that the current findings be replicated in more severe samples (e.g., clinical and forensic). A second limitation relates to the fact that arousal level/emotional reactivity was not directly measured. A more direct probe of biological variables through skin conductance or fMRI would therefore improve on the current study design. Finally, the post-hoc nature of the regression analysis should be born in mind when interpreting the moderation effect. Future research is necessary to evaluate the robustness with which HIA may limit expression of emotional underarousal in high CP children.

## Conclusions

Despite these limitations, the current study makes an important contribution in being one of the few studies to explicitly revisit Lynam's algorithm for identifying the fledgling psychopath, and by suggesting that hyperactivity may not actually facilitate emergence of core features of psychopathy in youth. While our findings do not directly speak to treatment issues for child psychopathy--an area which remains understudied--they do contribute to comparatively limited research on the factors that may dampen or promote the development of psychopathy. As such, these findings may be helpfully incorporated in the clinical conceptualization of HIA, CP, and psychopathy and how these disorders may be distinguished from one another.

## Appendix 1: Footnotes

^1 ^All but one of the contrasts (CP-only v. HIA-CP) survived controls for gender and IQ.

^2 ^All but one of the contrasts (HIA-only v. HIA-CP for CU traits) survived controls for gender and IQ.

^3 ^The interaction term remained significant with inclusion of gender and IQ as covariates.

^4 ^Sevecke et al. [53] did find that ADHD uniquely predicted psychopathic traits (including affective traits) over and above CD for girls, however.

## Competing interests

Neither of the authors has received reimbursements, fees, funding, or salary from an organization that may in any way gain or lose financially from the publication of this manuscript, either now or in the future. Neither of the authors holds any stocks or shares in an organization that may in any way gain or lose financially from the publication of this manuscript, either now or in the future. Neither of the authors holds or are currently applying for any patents relating to the content of the manuscript. There are no nonfinancial competing interests (political, personal, religious, ideological, academic, intellectual, commercial or any other) to declare in relation to this manuscript.

## Authors' contributions

As principal investigator on this study, CS collected the data. She also consulted in the analyses and assisted drafting of the paper. JDM took the lead on drafting the paper and carried out the analyses. Both authors read and approved the final manuscript.
